# Investigation of the Pattern of the Hemodynamic Response as Measured by Functional Near-Infrared Spectroscopy (fNIRS) Studies in Newborns, Less Than a Month Old: A Systematic Review

**DOI:** 10.3389/fnhum.2018.00371

**Published:** 2018-10-02

**Authors:** Isabel de Roever, Gemma Bale, Subhabrata Mitra, Judith Meek, Nicola J. Robertson, Ilias Tachtsidis

**Affiliations:** ^1^Department of Medical Physics and Biomedical Engineering, University College London, London, United Kingdom; ^2^Department of Neonatology, Institute for Women's Health, University College London, London, United Kingdom

**Keywords:** near-infrared spectroscopy, functional activation, newborns, infant, neurovascular coupling, hemodynamic response, stimulus, brain activity

## Abstract

It has been 20 years since functional near-infrared spectroscopy (fNIRS) was first used to investigate the evoked hemodynamic response to a stimulus in newborns. The hemodynamic response to functional activation is well-established in adults, with an observed increase in concentration change of oxygenated hemoglobin (Δ[HbO_2_]) and decrease in deoxygenated hemoglobin (Δ[HHb]). However, functional studies in newborns have revealed a mixed response, particularly with Δ[HHb] where an inconsistent change in direction is observed. The reason for this heterogeneity is unknown, with potential explanations arising from differing physiology in the developing brain, or differences in instrumentation or methodology. The aim of this review is to collate the findings from studies that have employed fNIRS to monitor cerebral hemodynamics in term newborn infants aged 1 day−1 month. A total of 46 eligible studies were identified; some studies investigated more than one stimulus type, resulting in a total of 51 reported results. The NIRS parameters reported varied across studies with 50/51 cases reporting Δ[HbO_2_], 39/51 reporting Δ[HHb], and 13/51 reporting total hemoglobin concentration Δ[HbT] (Δ[HbO_2_] + Δ[HHb]). However, of the 39 cases reporting Δ[HHb] in graphs or tables, only 24 studies explicitly discussed the response (i.e., direction of change) of this variable. In the studies where the fNIRS responses were discussed, 46/51 cases observed an increase in Δ[HbO_2_], 7/51 observed an increase or varied Δ[HHb], and 2/51 reported a varied or negative Δ[HbT]. An increase in Δ[HbO_2_] and decrease or no change in Δ[HHb] was observed in 15 studies. By reviewing this body of literature, we have identified that the majority of research articles reported an increase in Δ[HbO_2_] across various functional tasks and did not report the response of Δ[HHb]. Confirming the normal, healthy hemodynamic response in newborns will allow identification of unhealthy patterns and their association to normal neurodevelopment.

## Introduction

Monitoring brain activity in newborn populations up to 1 month old is of increasing interest not only for neuroscientists and psychologists who want to develop a deeper understanding of the brain and its development, but also for clinicians to derive prognostic markers of neurodevelopment following perinatal brain injury [such as hypoxic-ischaemic encephalopathy (HIE)].

Since the first functional near-infrared spectroscopy (fNIRS) study in newborns in 1998 by Meek et al. (1998) there have been a number of studies using this technique to investigate brain function and development in newborns, as well as older infants and children (Lloyd-Fox et al., 2010; McDonald and Perdue, 2018).

fNIRS is a non-invasive, non-ionizing neuromonitoring technique. It relies on the fact that tissue is relatively transparent to light in the near-infrared region (650–1,000 nm), and oxygenated- (HbO_2_) and deoxygenated- (HHb) hemoglobin are strong absorbers in this region. Similar to functional magnetic resonance imaging (fMRI), fNIRS is able to detect functional activity indirectly via detection of hemodynamic changes. Whilst fMRI is able to detect changes in HHb, fNIRS has the ability to differentiate between HbO_2_ and HHb, providing additional hemodynamic and oxygenation information.

One of the main advantages of the technique comes from the practical aspects of fNIRS devices: mainly the instruments can be deployed with relative ease, making it easy to use in natural settings without the need for large and bulky equipment. Although the spatial resolution is poorer compared to fMRI, the technique is less susceptible to movement artifacts, reducing the need for subjects to remain very still or be sedated. It is therefore an appropriate tool to study the newborn brain.

fNIRS monitors brain hemodynamic changes indirectly via measuring the concentration changes Δ[HbO_2_] and Δ[HHb]; these are secondary to the changes in local neural activity that lead to a corresponding oversupply of cerebral blood flow (CBF) to the functional localized area. Neuronal activation requires energy; a normal physiological response is an increase in CBF that overcompensates the tissue's energy demand. This leads to a decrease in Δ[HHb] and increase in Δ[HbO_2_], as HHb is flushed away while HbO_2_ flows in. This coupling between neural activity and CBF is known as neurovascular coupling (NVC).

The typical hemodynamic response in adults has been well-established, demonstrating an increase in Δ[HbO_2_] and total hemoglobin concentration Δ[HbT] (= Δ[HbO_2_] + Δ[HHb]) and decrease in Δ[HHb], with reproducible and consistent results at the group level (Plichta et al., 2006, 2007). However, studies in newborns have demonstrated a mixed hemodynamic response compared to adult studies, with an increase in Δ[HHb] also observed. It has been suggested that the variation in response may be due to the differing physiology in newborns, where components related to neurovascular coupling are still developing, and systemic blood pressure changes occurring during the stimuli confounding the hemodynamic response (Kozberg and Hillman, 2016). Likewise, conflicting results could be due to inter-study differences, where differing study paradigms, such as the method or type of stimulation, may affect results. Differences in instrumentation used and the waking state of the newborn (whether awake, asleep, or sedated), may also contribute to the variation observed.

**Figure 1:**
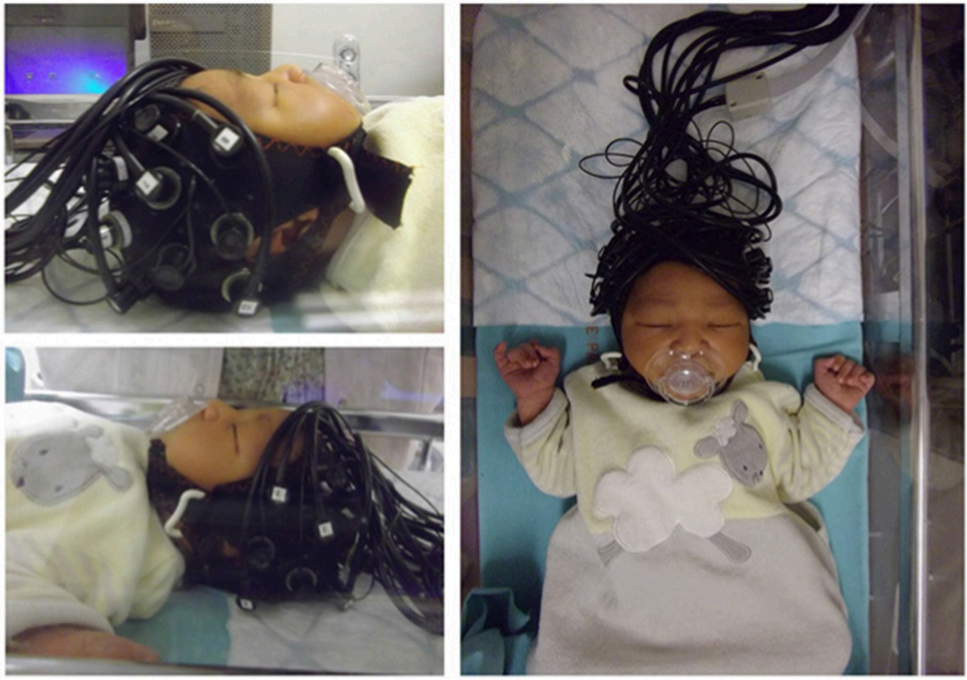
Example of NIRS headgear on a newborn to monitor functional activation. Reproduced from Bouchon et al. (2015) with permission.

**Figure 2:**
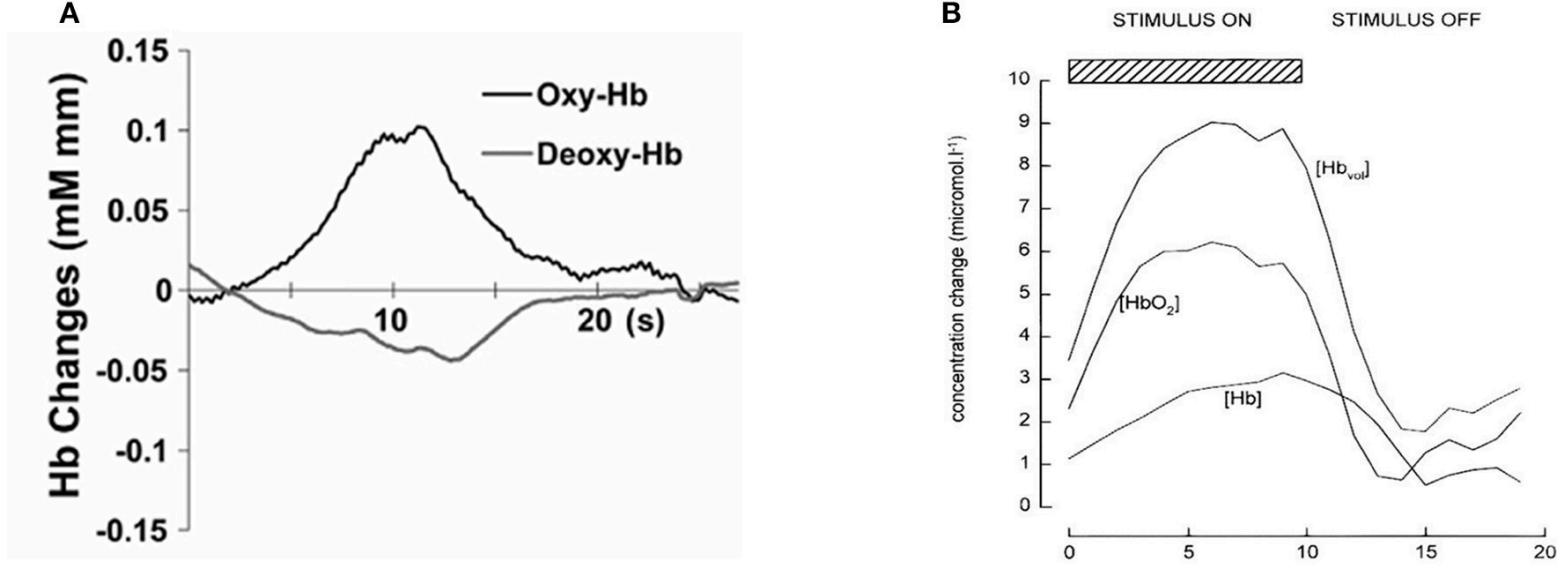
Example of functional responses in newborns. **(A)** Response for an asleep newborn in response to auditory stimulation. Shows an increase Δ[HbO_2_] (labeled as oxy-Hb) and a slight decrease in Δ[HHb] (labeled as deoxy-Hb). Reproduced from Arimitsu et al. (2018) with permission. **(B)** Response for an awake newborn in response to visual stimulation. Shows an increase in Δ[HbO_2_], Δ[HbT] (labeled as [Hb_vol_]), and Δ[HHb] (labeled as [Hb]). Reproduced from Meek et al. (1998) with permission.

**Figure 3:**
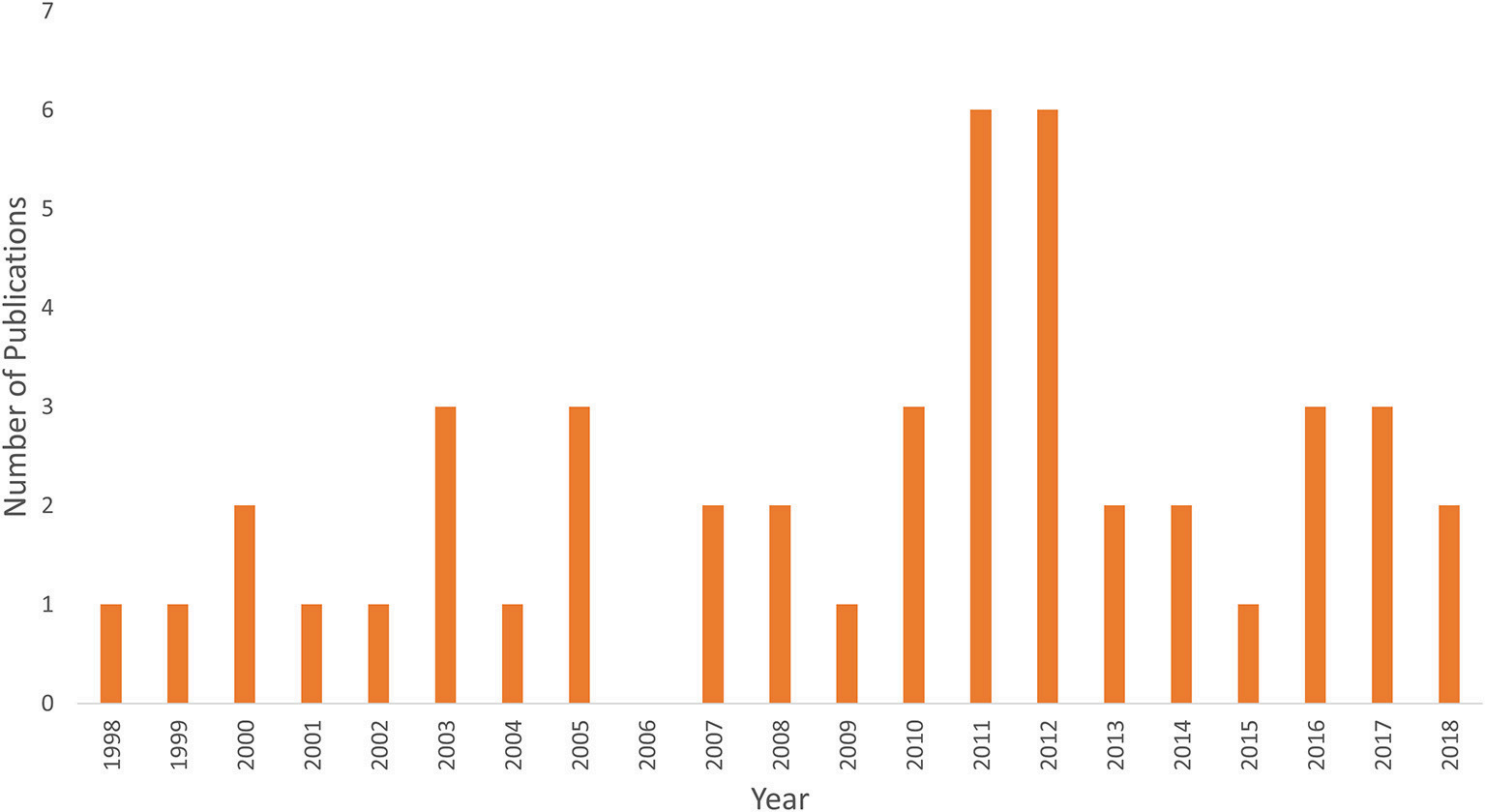
Graph showing the number of publications using fNIRS in term newborns <1 month of age since the first study in 1998.

Figure 1 shows an example of fNIRS instrumentation on a newborn; in this case, the light sources and detectors are placed in a cap, which is then placed on the newborn's head. Figure 2 shows some examples of hemodynamic responses observed in functional studies in newborns, where (a) shows a response similar to a typical adult response and (b) shows an inverted Δ[HHb] response. Figure 3 presents the number of fNIRS related publications in term neonates over the last 20 years.

Previous review papers have included summarizing the challenges and practicalities in performing fNIRS in infants (Lloyd-Fox et al., 2010) and investigating the inverted hemodynamic response in infants up to around 24 months of age with respect to the experimental design and stimulus complexity (Issard and Gervain, 2018).

The purpose of this review is to investigate the pattern of the hemodynamic response of healthy, term newborns to a stimulus, within a tightly controlled age range from birth to 1 month of age. This is a sensitive age range, where rapid growth and developmental changes are occurring in the brain, and is also an age of particular interest as it is a period when the newborn is at risk of significant brain injury. For example, HIE occurs in 1–2 per 1,000 live births (James and Patel, 2014) and is associated with neurodevelopmental impairment and mortality, and is an active area of research. It is therefore important to understand the typical response of the healthy newborn brain such that future work is able to identify abnormal response patterns associated with brain injury in this cohort.

## Methods

**Figure 4:**
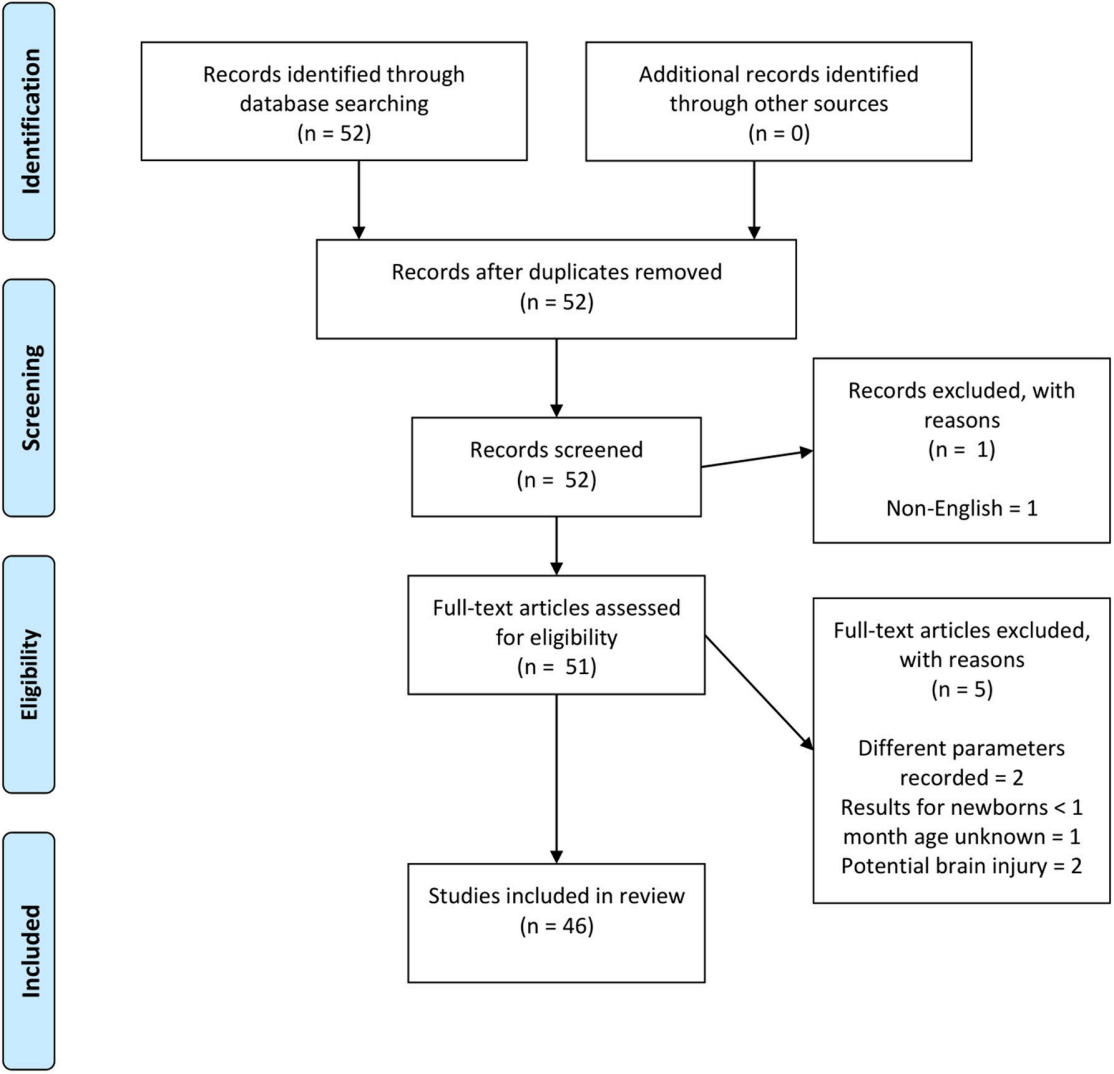
PRISMA chart showing papers identified, eligibility, and inclusion in this review paper. Note, two papers (Abboub et al., 2016; Ferry et al., 2016) were later identified that were not flagged using the defined search criteria. These are cited here but not included in the final analysis.

The focus of the review was to look at whether the fNIRS-measured hemodynamic response of healthy newborns <1 month of age compared to the expected hemodynamic response of an increase in Δ[HbO_2_] and decrease in Δ[HHb] and, if not, whether the variability of the hemodynamic response can be explained. Therefore, papers were identified using PubMed and Scopus, searching for a combination of keywords including (near-infrared spectroscopy | near infrared | optical | tomography) and (neonate | newborn) and (functional activation | activation | evoked response | response). The PRISMA chart for the selection of papers included in this review is shown in Figure 4. Papers were rejected if different parameters to Δ[HbO_2_], Δ[HHb], and Δ[HbT] were reported, if results from term newborns <30 days old could not be extracted from a larger cohort outside this target range, or if studies were performed on newborns with suspected brain injury, such as HIE.

## Results

### Patterns of hemodynamic response to different functional protocols

A total of 46 studies using fNIRS in neonates were identified, with a total of 51 sets of results arising from some studies investigating more than one stimulus type. A summary of the studies included in this review is presented in Table 1 and includes the cortical area of interest, stimulus type and hemodynamic response. The majority of studies were on healthy newborns. Some studies monitored newborns with potential pathology (marked with an asterisk in Table 1); however, these studies only included newborns with pathological conditions unrelated to cerebral function and with no congenital abnormalities. One study investigated newborns with HIE alongside a control group (Chen et al., 2002); only the results from the control group are presented here. Similarly, some studies looked at both term and preterm newborns (Isobe et al., 2001; Ozawa et al., 2011a; Naoi et al., 2013; Carlier-Torres et al., 2014; Frie et al., 2017; Arimitsu et al., 2018) or included subjects older than 30 days (Meek et al., 1998); the results presented here only include the responses from the term infants <30 days old.

**Table 1:** Table of literature review of fNIRS studies on healthy term newborns <1 month old, GA = gestational age.

**First author (year)**	**Participants**	**Instrumentation**	**Cortical area of interest**	**Experimental protocol**	**Significant results**		
					**HbO_2_**	**HHb**	**HbT**
Meek et al., 1998^*^	Included 3 awake term newborns, 3 days−3 weeks	NIRO 500, Hamamatsu	Occipital	Visual. Checkerboard 5 Hz pattern reversal, 10 s on, 10 s off	+	+	+
Sakatani et al., 1999	28 newborns, mean 3.1 days	NIRO 500, Hamamatsu	Frontal	Auditory. 60 dB piano music, 10 min on, 10 min off	+	Varied	+
Hoshi et al., 2000	7 asleep newborns, 4–5 days	OMM-100, Shimadzu	Occipital	Visual. 10 Hz flashing light, 30 s on, off until baseline	+	Varied	+
Bartocci et al., 2000	23 awake newborns, 1–8 days	NIRO 300, Hamamatsu	Frontal	Olfactory. Two odors: vanilla and mother's colostrum. Control: distilled water. 30 s on, 2 min baseline	+	Not reported	+
Isobe et al., 2001	Included 2 sedated term newborns, 5 days and 9 days	Hitachi	Parieto-temporal	Motor. Passive knee movement, 15 s on, 30 s off	+	–	Not reported
Chen et al., 2002	Included 20 healthy term newborns, 1–3 days	NIRO 500, Hamamatsu	Frontal	Auditory. 60 dB piano music, 10 min on, 10 min off	+	Varied	+
Peña et al., 2003	12 asleep newborns, 2–5 days	ETG-100, Hitachi	Temporal, fronto-parietal	Auditory. Normal speech, backwards speech, silence. 15 s on, 25–35 s off	Not reported	Not reported	+
Taga et al., 2003	25 newborns-good data from 16 asleep newborns, GA 38–42 weeks	Hitachi	Occipital and frontal	Visual. 14 Hz flashing light, 3 s on, 20 s off	+	Varied	Not reported
Nissilä et al., 2003	10 newborns, 0.5–4 days	In-house built, Helsinki	Temporal and parietal	Auditory. 1 kHz tone, 30 s on, 30 s off. Somatosensory.Touching heel, 30 s on, 30 s off	Tactile: +; Auditory: Not discussed but shown	Tactile: Not discussed but shown; Auditory: Not discussed but shown	Not reported
Nissila et al., 2004	10 asleep and awake newborns, mean 1.4 days	In-house built, Helsinki	Temporal	Auditory. Sinusoidal tones. 100 ms on, 25 s off	+	– or no change	Not reported
Haensse et al., 2005	1 newborn, GA 38 weeks	In-house built, Zurich	Parietal	Sensory. Vibration to palm, 20 s on, 10 s rest	+	–	Not reported
Kotilahti et al., 2005	20 awake and asleep newborns, 1–3 days	In-house built, Helsinki	Temporal	Auditory. Sinusoidal tones, 60 dB. 5 s on, 25 s off	+	Not discussed but shown	Not reported
Kusaka et al., 2005^*^	5 asleep newborns, 9 days−16 weeks	OMM-2000, Shimadzu	Occipital	Visual. Stroboscopic white flashing light, 8 Hz onto eyelids. 15 s on, 45 s off	–	+	–
Saito et al., 2007a	20 asleep newborns, 2–9 days	NIRO 200, Hamamatsu	Frontal	Auditory. Infant-directed and adult-directed speech, 15–28 s on, off until return to baseline	+	Not reported	Not reported
Saito et al., 2007b	20 asleep newborns, 1–9 days	NIRO 200, Hamamatsu	Frontal	Auditory. 60–70 dB normal, pitched and flat speech, 30 s on, 60 s off	+	Not reported	Not reported
Karen et al., 2008	20 asleep newborns, median age 5.5 days	In-house built, MCP-II	Occipital	Visual. Flashing red LEDs, 0.5–1 Hz, 20 s on, 20 s off	+	–	+
Gervain et al., 2008	22 newborns, mean age 3.14 days 22 newborns, mean age 2.86 days	ETG-4000, Hitachi	Frontal, temporal	Auditory. Language repetition sequences, 18 s on, 25–35 s off	+	Not discussed but shown	Not reported
Telkemeyer et al., 2009	34 newborns, 2–6 days	Omniat Tissue Oxymeter, ISS	Frontotemporal to tempoparietal	Auditory. Tonal recording 70 dB, 9 s on, 1–12 s off (mean 4.1 s)	+	–	Not reported
Aoyama et al., 2010	34 asleep newborns, 2–9 days	NIRO-200, Hamamatsu	Frontal	Olfactory. Two odors: breast milk, artificial milk. 30 s on, 60 s off	+	Not reported	Not reported
Kotilahti et al., 2010	13 asleep newborns, 1–4 days	In-house built, Helsinki 2	Temporal	Auditory. Infant-directed speech and piano music, 60 dB. 5 s on, 15 s off	Varied	For positive HbO_2_, HHb-	Varied
Liao et al., 2010	11 newborns, 1–3 days	In-house built, Washington	Occipital	Visual. Counterphase checkerboard pattern, 10 s on, 20 s off	+	–	+
Benavides-Varela et al., 2011	12 newborns, 1–5 days	ETG-4000, Hitachi	Frontal, parietal, temporal	Auditory. Consonant (C) Vowel (V) CVCV speech, 70 dB. 10 s on, 25–35 s off	+	Not discussed but shown	Not reported
Arimitsu et al., 2011	17 asleep newborns, 3–8 days	ETG-4000, Hitachi	Frontal, parietal, temporal	Auditory. Phonemic and prosodic speech, 67 dB, 15 s on, 15 s off	+	Not discussed but shown	Not reported
May et al., 2011	20 newborns, 1–3 days	ETG-4000, Hitachi	Frontal, parietal, temporal	Auditory. Forwards and backwards speech, 70–75 dB, 18–20 s on, 25–35 s off	+	Not discussed but shown	Not reported
Minagawa-Kawai et al., 2011	38 asleep newborns, 1–5 days	NTS Optical Imaging System, UCL	Temporal, frontal	Auditory. Tone patterns 80 dB, 10 s on, 8–14 s off	+	Not discussed but shown	Not reported
Ozawa et al., 2011a^*^	40 newborns, 4–6 days	NIRO 200, Hamamatsu	Frontal	Pain. Blood sampling on dorsum of hand, monitoring 5 minutes before, then during blood sampling procedure	+	Not reported	Not reported
Ozawa et al., 2011b^*^	Included 50 term newborns, 4–6 days	NIRO 200, Hamamatsu	Frontal	Pain. Skin-breaking, monitoring 5 min baseline and during procedure	+	Not reported	Not reported
Benavides-Varela et al., 2012	44 newborns, mean 2.5 days	ETG-4000, Hitachi	Frontal, temporal, parietal	Auditory. Consonant (C)/Vowel (V) CVCV words, 70 dB, 10 s on, 25–35 s off	+	No change	Not reported
Biallas et al., 2012	14 asleep newborns, mean age 2.1 days	In-house built, Zurich 2	Occipital	Visual. Light flashing 0.5 Hz, 20 s on, 12–32 s off	Varied	Not reported	Not reported
Gervain et al., 2012	22 newborns, 1–3 days	ETG-4000, Hitachi	Frontal, temporal	Auditory. Short repetition sequences (speech), 18 s on, 25–35 s off	+	–	Not reported
Liao et al., 2012	11 newborns, 1–2 days	In-house built, Washington	Occipital	Visual. Counterphase checkerboard pattern, 10 s on, 20 s off	+	–	+
Sato et al., 2012	17 newborns, 1–7 days	Modified ETG-7000, Hitachi	Whole-head	Auditory. Forwards and backwards speech, 62–65 dB, 10 s on, 20–30 s off	+	–	+
Shibata et al., 2012	10 asleep newborns, 2–9 days	Modified FOIRE-3000/16, Shimadzu	Parietal, temporal, occipital	Tactile. Vibration to palm, 10 s on, 25–30 s off. Auditory. Speech and music audio, 5 s on, 25–30 s off. Visual. Flashing light at 8 and 20 Hz, 5 s on, 25–30 s off	Tactile: + Auditory: + Visual: +	Not reported	Not reported
Bembich et al., 2013	30 newborns, 3 days	ETG-4000, Hitachi	Parietal, temporal, frontal	Pain. 10 s before, during stimulus, 25 s after	+	Not discussed but shown	Not reported
Naoi et al., 2013	Included 29 asleep term newborns, mean 4.7 days	ETG-7000, Hitachi	Frontal, temporal, parietal, occipital	Infant-directed speech, adult-directed speech and pink noise (control), 62 dB. 20 s on, 20 s off	+	Not discussed but shown	Not reported
Carlier-Torres et al., 2014	Included 13 asleep term newborns, mean GA 38 ± 1 weeks	ETG-4000, Hitachi	Temporal	Auditory. Speech (consonant, vowel sounds), 76 dB, 15 s on, 20–30 s off	+	–	Not reported
Cristia et al., 2014	40 newborns, 1–6 days	NTS Optical Imaging System, UCL	Temporal, frontal, temporoparietal	Auditory. Native and non-native speech and macaque sounds, 75 dB, 10 s on, 8–16 s off	+	Not discussed but shown	Not reported
Bouchon et al., 2015	24 asleep newborns, 1–3 days	NIRScout 816, NIRx	Temporal, frontal	Auditory. Speech (repetitive consonant vowel sounds). 9.9–10.9 s on, 20–25 s off	+	Not discussed but shown	Not reported
Gervain et al., 2016	22 newborns, 1–3 days	ETG-4000, Hitachi	Temporal	Auditory. Water sounds. 18 s on, 25–35 s off	+	–	Not reported
Vannasing et al., 2016	27 asleep newborns, 1 day	Imagent Oxymeter, ISS	Temporal	Auditory. Forwards and backwards speech. 64–76 dB, 20 s on, 40 s off	+	Not discussed but shown	Not reported
Verriotis et al., 2016	36 newborns, 2–9 days	NIRO 200NX, Hamamatsu	Occipito-parietal	Somatosensory. Noxious: heel lance, innocuous: tactile. Auditory: click of lancet. 30 s baseline, stimuli, 30 s baseline	Tactile: + Pain: + Auditory: +	Tactile: no change Pain: Varied Auditory: no change	Pain: +
Frie et al., 2017^*^	Included 17 term newborns, 1–3 days	NIRScout, NIRx	Frontal, parietal	Olfactory. Three odors soaked in cotton bud held 1 cm below nose: pure handcleaner, diluted handcleaner, adhesive remover. Control: water. 10 s on, 2 min off	+	–	Not reported
Issard and Gervain, 2017	59 newborns, 1–4 days	NIRScout 816, NIRx	Fronto-temporal, temporal, tempero-parietal	Auditory. Infant-directed speech (combination of syllables, compressed and non-compressed). 17–19 s on, 26–35 s off	+	Not discussed but shown	Not reported
Zhang et al., 2017	18 newborns, 2–6 days	NIRScout 1624, NIRx	Frontal, temporal	Auditory. Four emotions presented through speech: fear, anger, happiness and neutral. 55–60 dB. 15 s on, 14–16 s off	+	Not discussed but shown	Not reported
Arimitsu et al., 2018^*^	Included 20 asleep term newborns, median 9 days	ETG-4000, Hitachi	Temporal	Auditory. Three words with prosodic and phonemic distinctions, 67 dB. 15 s on, 15 s off	+	–	Not reported
May et al., 2018	24 newborns, 0–3 days	ETG-4000, Hitachi	Temporal	Auditory. Forwards and backwards speech. 15 s on, 25–35 s off.	+	Not discussed but shown	Not reported

* *Neonates with potential pathology unrelated to cerebral function*.

It should be noted that not all papers reported all three NIRS parameters, Δ[HbO_2_], Δ[HHb], and Δ[HbT]. In some papers, the measurement of Δ[HHb] was reported in graphs or tables but the direction of the response was not explicitly analyzed and discussed, with discussion often centered around the direction of Δ[HbO_2_].

**Figure 5:**
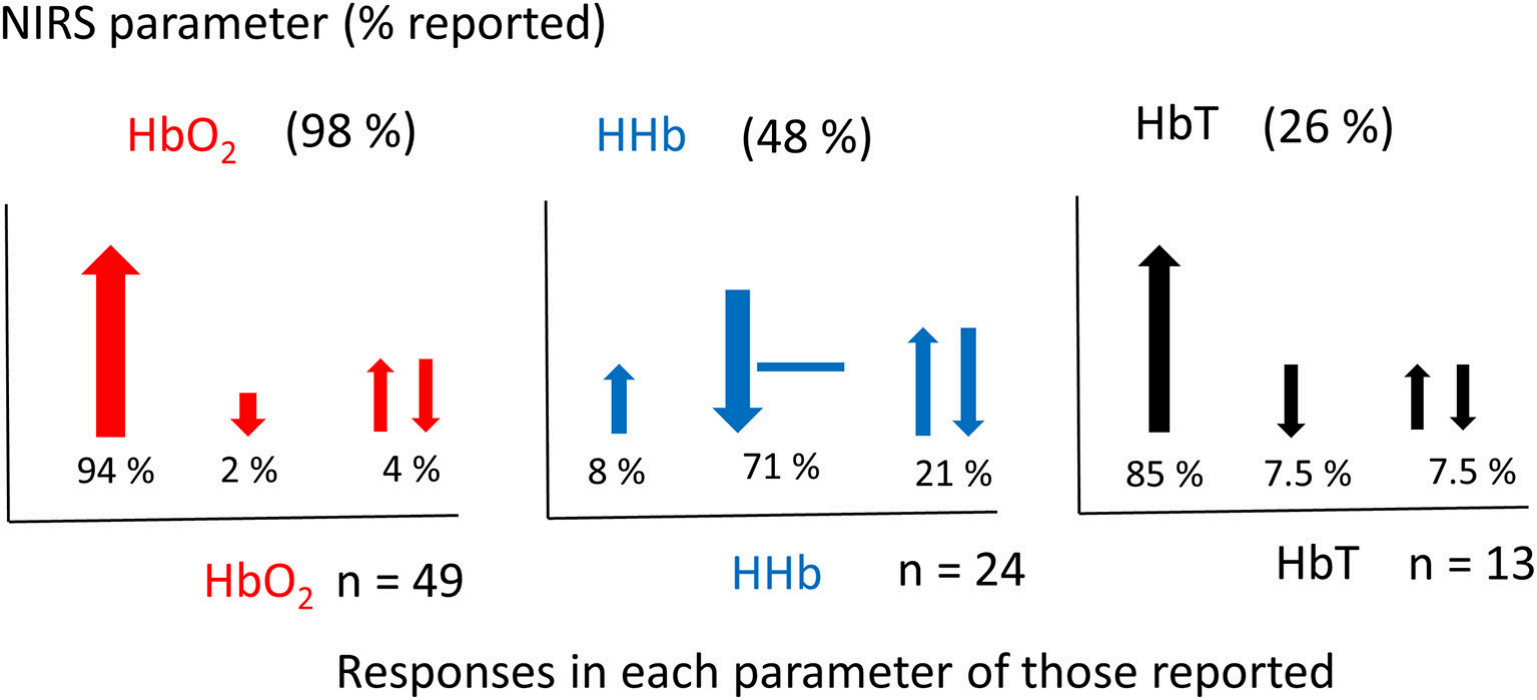
Graphs showing the percentage of NIRS parameters reported in fNIRS studies on newborns (above) (a total set of 51 responses from 46 studies were identified) and the corresponding responses of those reported (below). Changes in [HbO_2_] shown in red, [HHb] shown in blue and [HbT] shown in black. The total number of studies (*n*) reporting each variable is stated next to each graph. Directions of responses are indicated with an arrow or a line if no change; responses with a double arrow indicate a mixed response (both positive and negative changes observed). Size of arrows correspond to occurrence.

**Figure 6:**
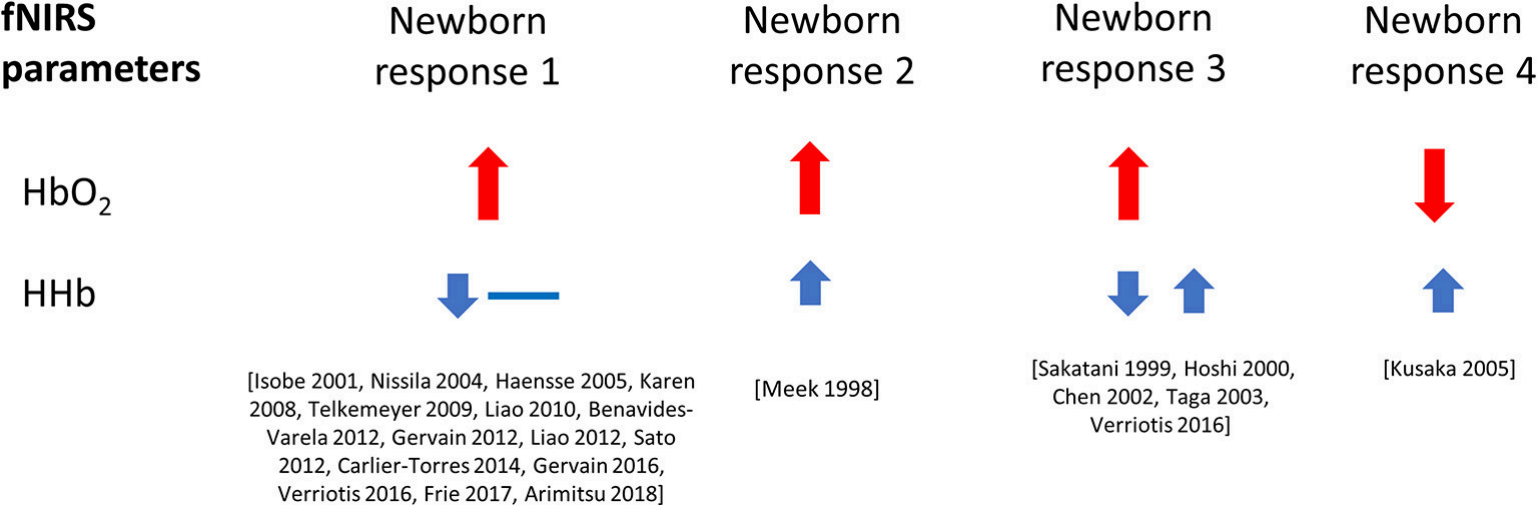
Haemodynamic responses to functional activation observed in newborns, with changes in Δ[HbO_2_] shown in red and Δ[HHb] shown in blue. Data is taken from literature identified in this review paper, and included above only when both variables, Δ[HbO_2_] and Δ[HHb], have been reported and discussed. The size of the arrows (small and large) relate to the magnitude of the response. Responses with a double arrow indicate a mixed response (both positive and negative changes observed).

Figure 5 shows the percentage of studies that reported the different NIRS parameters, and of those parameters that were reported, what the observed response was (this does not include responses where only the measurements are presented but not explicitly discussed). From the 51 results reported, 49 discussed Δ[HbO_2_], 24 discussed Δ[HHb], and 13 discussed Δ[HbT]. A summary of the Δ[HbO_2_] and Δ[HHb] reported responses is shown in Figure 6. An increase in Δ[HbO_2_] and decrease or no change in Δ[HHb] was observed in 15 studies.

**Figure 7:**
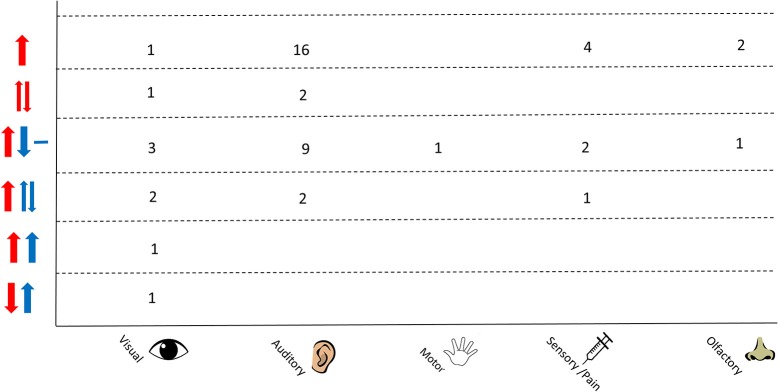
Chart showing observed responses separated by stimulus type. Number of studies showing observed response is shown. Directions of arrow indicate whether an increase, decrease, varied response or no change was seen in the NIRS parameter. Δ[HbO_2_] is shown in red and Δ[HHb] in blue.

An overview of the responses as separated by stimulus type is shown in Figure 7. A variation in responses is seen in studies using auditory stimuli which may be due to the higher number of studies that employ this as a stimulus. The most common response in this protocol is an increase in Δ[HbO_2_] and decrease in Δ[HHb] with nine studies reporting this response; two studies observed a varied Δ[HHb] response and two studies observed a varied Δ[HbO_2_]. Studies using a visual stimulus also show a variety of responses, with Δ[HHb] showing variability or an increase in four out of nine studies. A varied Δ[HHb] is also observed in response to a pain stimulus, as identified in one study, with two sensory studies reporting an increase in Δ[HbO_2_] and decrease in Δ[HHb].

The study design is an important consideration when looking at fNIRS data. The type of stimulus used to evoke a cerebral response produces differing responses, with variation in the size of the response and temporal profile (Kamran et al., 2015), which may be due to different capillary transit times across cortices (Jasdzewski et al., 2003). Hence, results presented in the next section are separated by functional task (auditory, visual, motor or sensory/pain and olfactory) for ease of comparison.

#### Visual stimulation

Nine fNIRS studies used visual stimulation. Visual stimuli generally used either a checkerboard pattern (Meek et al., 1998; Liao et al., 2010, 2012) or a stroboscopic light (Hoshi et al., 2000; Taga et al., 2003; Kusaka et al., 2005; Karen et al., 2008; Biallas et al., 2012; Shibata et al., 2012). All studies monitored the occipital lobe, with some studies additionally monitoring the frontal lobe (Taga et al., 2003) and temporal and parietal lobe (Shibata et al., 2012).

Two of the three studies employing a checkerboard pattern observed an increase in Δ[HbO_2_] and decrease in Δ[HHb] (Liao et al., 2010, 2012), with the remaining study observing an increase in Δ[HbO_2_] and increase in Δ[HHb] (Meek et al., 1998). All three studies found the time-course of Δ[HbO_2_] to be similar to that of adults.

The remaining six studies employed a stroboscopic light to elicit a visual response. The studies show similar results for Δ[HbO_2_], with most studies observing a positive change in this parameter apart from Kusaka et al. (2005) who observed a negative Δ[HbO_2_] and Biallas et al. (2012) who observed both positive and negative Δ[HbO_2_]. Kusaka et al. (2005) additionally observed an increase in Δ[HHb]. Two studies did not report the Δ[HHb] results (Biallas et al., 2012; Shibata et al., 2012), two studies reported a varied response (both positive and negative; Hoshi et al., 2000; Taga et al., 2003) and one study reported a negative Δ[HHb] (Karen et al., 2008). Hoshi et al. (2000) also noted a relatively slow recovery of the fNIRS parameters after the end of the stimulus compared to rapid recoveries previously reported in adults.

#### Auditory stimulation

Thirty studies on newborns used an auditory stimulus; this is the most commonly investigated stimulus using fNIRS in newborns, with a large number of papers (19 studies) investigating language and memory development in the newborn brain. The majority of studies (25/30) monitored the temporal lobe, otherwise the frontal lobe was monitored instead (Sakatani et al., 1999; Chen et al., 2002; Saito et al., 2007a,b); some studies monitored other brain regions in addition to the temporal and frontal lobes [frontoparietal (Peña et al., 2003), temporoparietal (Telkemeyer et al., 2009; Cristia et al., 2014; Issard and Gervain, 2017), parietal (Arimitsu et al., 2011; Benavides-Varela et al., 2011, 2012; May et al., 2011; Molavi et al., 2012), whole-head (Sato et al., 2012; Naoi et al., 2013)]. Paradigms consisted of either music or tonal sounds, or a variation of speech, such as non-native language or backwards speech.

Four studies presented music as an auditory stimulus reporting a positive change in Δ[HbO_2_] (Sakatani et al., 1999; Chen et al., 2002; Kotilahti et al., 2010; Shibata et al., 2012) with two studies (Sakatani et al., 1999; Chen et al., 2002) also reporting a varied Δ[HHb] and one study (Shibata et al., 2012) not reporting Δ[HHb]. Kotilahti et al. (2010) observed a varied Δ[HbO_2_]; for positive Δ[HbO_2_] cases, they observed a negative Δ[HHb]. They also reported this same response when using a speech stimulus (see below).

Five studies used a tonal sound to evoke an auditory response. A positive Δ[HbO_2_] was seen in most of the studies (Nissila et al., 2004; Kotilahti et al., 2005; Telkemeyer et al., 2009; Minagawa-Kawai et al., 2011) apart from Nissilä et al. (2003) where the direction of the response for the auditory stimulation is not explicitly discussed. A negative or no change in Δ[HHb] is reported in two studies (Nissila et al., 2004; Telkemeyer et al., 2009). Telkemeyer et al. (2009) identified a vascular response time-course similar to the well-established adult vascular response dynamics. Kotilahti et al. (2005) additionally noted the latency of the maximum HbO_2_ response decreased with gestational age, with a mean latency of 9.6 ± 2.2 s. This was for a cohort of twenty term infants with gestational ages between 38.7 and 42.3 weeks.

Nineteen studies used speech as an auditory stimulus, with seventeen studies reporting a positive change in Δ[HbO_2_] (Saito et al., 2007a,b; Gervain et al., 2008, 2012; Arimitsu et al., 2011; Benavides-Varela et al., 2011; May et al., 2011, 2018; Sato et al., 2012; Naoi et al., 2013; Carlier-Torres et al., 2014; Cristia et al., 2014; Bouchon et al., 2015; Vannasing et al., 2016; Issard and Gervain, 2017; Zhang et al., 2017). One study reported a varied Δ[HbO_2_] (Kotilahti et al., 2010) and one study did not report Δ[HbO_2_] but instead presented a positive Δ[HbT] response (Peña et al., 2003). A negative or no change in Δ[HHb] was observed in five studies (Kotilahti et al., 2010; Gervain et al., 2012; Sato et al., 2012; Carlier-Torres et al., 2014; Arimitsu et al., 2018). It is noted that some studies did not explicitly state the direction of Δ[HbO_2_] but instead discussed “activation” (Gervain et al., 2008, 2016; Cristia et al., 2014; Bouchon et al., 2015; Zhang et al., 2017; May et al., 2018); in these cases, the graphs were visually inspected and a positive HbO_2_ response identified as indicative of activation.

Additionally, some studies performed more than one task as an auditory stimulus, however, only one response per stimulus-type is reported here. For example, Benavides-Varela et al. (2012) identified an increase in Δ[HbO_2_] when presenting novel vowel sounds and a decrease in Δ[HbO_2_] when presenting novel consonant sounds. They interpret this as a privilege to vowel information in the newborn and hence only the increase in Δ[HbO_2_] is reported here. Additionally, Issard and Gervain (2017) used auditory stimuli consisting of normal speech and compressed speech. They identified a positive HbO_2_ response with normal speech but an inverse HbO_2_ response to highly compressed speech; they considered this inverse response as a deactivation and hence this deactivated response is not recorded here. Three further studies presented multiple auditory tasks (Saito et al., 2007a,b; May et al., 2011); in these cases, only the positive HbO_2_ response was interpreted as a functional response as identified from the discussion of the results and only these responses are reported here.

Two other auditory paradigms were identified that do not fit into the categories above. One used water sounds and identified a positive Δ[HbO_2_] and negative Δ[HHb] (Gervain et al., 2016). The other used the sound of a heel lance as a control for a noxious task involving a heel lance and observed a positive change in Δ[HbO_2_] and no change in Δ[HHb] (Verriotis et al., 2016).

#### Motor or sensory/pain stimulation

Eleven studies have been performed in newborns using motor or sensory/pain stimulation. Stimuli consisted of passive knee or elbow movement (Isobe et al., 2001), vibration to hand or foot (Haensse et al., 2005; Shibata et al., 2012), tapping (Nasser et al., 2016; Verriotis et al., 2016), rubbing the foot (Nissilä et al., 2003), or breaking skin as part of standard clinical care (Ozawa et al., 2011a,b; Bembich et al., 2013; Verriotis et al., 2016). All studies monitored parietal or frontoparietal regions apart from two noxious studies where the frontal lobe was monitored (Ozawa et al., 2011a,b). Bembich et al. (2013) also monitored over the frontal and temporal regions. One study monitored newborns who were sedated (Isobe et al., 2001).

Ten studies showed a positive Δ[HbO_2_] response with one study not reporting Δ[HbO_2_] (they instead, they report cerebral blood volume calculated using Δ[HbT]^*^0.69/Δ[HHb]; Beken et al., 2014). Three studies displayed a negative or no change in Δ[HHb] (Isobe et al., 2001; Haensse et al., 2005; Verriotis et al., 2016) and four studies did not report Δ[HHb] results (Ozawa et al., 2011a,b; Shibata et al., 2012; Beken et al., 2014). Isobe et al. (2001) additionally noted a slower hemodynamic response compared to adults with a time-to-peak of 12 s in Δ[HbO_2_] and 19 s in Δ[HHb], compared to adult responses in a similar task of 6–8 s in Δ[HbO_2_] and 11–13 s in Δ[HHb], however, it should be noted that this study was performed on sedated newborns. Verriotis et al. (2016) identified a relatively short peak hemodynamic response of 2–4 s for Δ[HbO_2_] compared to previously reported peak latencies of 4–6 s in adults.

#### Olfactory stimulation

Three studies used an olfactory stimulus in newborns. All studies monitored the frontal lobe with one study additionally monitoring the parietal lobe (Frie et al., 2017). All three studies found a positive response of Δ[HbO_2_] with results of Δ[HHb] not being reported in two studies (Barton, 2000; Aoyama et al., 2010) and showing a negative response in one study (Frie et al., 2017).

## Discussion

### Interpretation of fNIRS studies in newborns

The majority of studies demonstrated an increase in Δ[HbO_2_] with most variation in response arising from Δ[HHb] (see Figure 6). This review separated the functional tasks by type, with results from auditory, visual, motor or sensory/pain and olfactory stimuli presented. No clear association between the reported response and stimulus type is observed.

It should be noted that studies using fMRI blood-oxygen-level dependent (BOLD), monitoring neuronal activation via hemodynamic changes, have also observed a heterogeneous response in the newborn brain. An early study by Born et al. (1998) looked at seventeen infants, 3 of whom were 4 weeks age or less and identified a mixed response to visual stimulation using a stroboscopic light during spontaneous sleep. A negative BOLD response, corresponding to an increase in Δ[HHb], suggests that the coupling between neural activity and vascular response is different in neonates compared to adults (Born et al., 1998). Some groups have observed positive BOLD responses in neonates in-line with a typical adult response. Arichi et al. (2009) studied six term infants (one sedated) using a somatosensory stimulus identifying a positive BOLD response. The authors hypothesize that negative BOLD signals may arise from analysis methods of fMRI data, where typically an adult-derived hemodynamic response function (HRF) model is used rather than an infant-HRF, which is likely to differ in shape. It has also been suggested that infant HRFs may differ in temporal profile as well and may be the cause of discrepancies seen in newborn BOLD responses (Seghier et al., 2006; Arichi et al., 2012).

Care should be taken when interpreting fMRI BOLD studies, as they are often conducted with infants under sedation to prevent movement artifacts. The effect of sedation on the hemodynamic response in human infants has not been investigated; however, in animal studies, sedation did not affect the response of HbO_2_ and HHb signals (Sharp et al., 2015).

A BOLD study by Arichi et al. looked at the BOLD response from two groups of term infants: one group sedated and one group unsedated. They identified no difference in global CBF between the two groups, suggesting the inverse BOLD response is not as a result of sedation (Arichi et al., 2012). However, other studies have suggested that sedation may alter the baseline CBF (Seghier et al., 2006); further investigation into different types of sedation and its effect on the hemodynamic response in newborns is needed. Additionally, since BOLD fMRI detects activated cortical regions via detection of a decrease in Δ[HHb], the presence of an increase in Δ[HHb] may be overlooked unless both an increase and decrease of the fMRI signal are considered.

Functional studies in adults have identified a typical hemodynamic response consisting of an increase in Δ[HbO_2_] that reaches a peak a few seconds before the peak decrease in Δ[HHb]. Some studies in newborns have shown a slower hemodynamic response compared to adults (~12–16 s peak latency compared to 4–6 s typical peak latency in adults during motor stimulation; Isobe et al., 2001). The slower response of the neonatal hemodynamic response compared to the adult response may be due to several factors such as sedation (for example, Isobe et al. (2001) monitored sedated newborns), a differing functional organization of the brain in newborns or on-going myelin and synapse development and hence a developing NVC mechanism (Kusaka et al., 2011). It has been suggested that myelination can effect the latency of the hemodynamic response, with increased myelination (such as in the adult brain) leading to a more synchronous synaptic activation (Harris et al., 2011). MRI studies have shown that visual and auditory sensory regions myelinate faster than motor regions (Welker and Patton, 2012), hence it is plausible that the latency of the hemodynamic response varies according to the stimulus type.

In contrast, Verriotis et al. (2016) identified a faster peak hemodynamic response of 2–4 s for Δ[HbO_2_] compared to peak latencies of 4–6 s in adults. They suggest this may be the result of differing stimulus durations but alternatively, be related to an immature vascular regulation in newborns that may result in reduced hyperemia and hence a shorter increase in Δ[HbO_2_].

The majority of studies reported here do not discuss the peak latencies. One study, however, did identify a relationship between the latency of the hemodynamic response to the gestational age of the subject, with significantly shorter latencies for infants with higher gestational age (Kotilahti et al., 2005), suggesting a variation in the NVC mechanism with age.

Some adult studies have additionally identified an initial dip in the hemodynamic response in the form of an immediate decrease in Δ[HbO_2_] and increase in Δ[HHb] after onset of the stimulus before the typical oxygen supply to oxygen utilization ratio is established. The origin of this dip is unknown but has been shown to be localized and may reflect localized neuronal activity (Zaidi et al., 2018). The majority of fNIRS studies on newborns do not observe this dip that is typical in the adult response. However, from visually inspecting the time-courses of the hemodynamic responses in this review, a dip was identified in some studies (Kotilahti et al., 2010; Liao et al., 2010, 2012; Arimitsu et al., 2011) although this was not discussed in any of the papers.

The following aspects need to be considered for understanding the fNIRS results: physiological mechanisms, study design, instrumentation and data analysis.

### Physiological mechanisms

Several physiological mechanisms have been hypothesized to explain the discrepancy in the newborn hemodynamic response, and in particular to explain the observed increase in Δ[HHb] reported in some studies (Meek et al., 1998; Sakatani et al., 1999; Hoshi et al., 2000; Chen et al., 2002; Taga et al., 2003; Kusaka et al., 2005; Verriotis et al., 2016).

Several studies observed an increase in Δ[HbO_2_] and decrease in Δ[HHb] similar to that observed in the adult brain, suggesting that NVC is intact and functioning in the newborn brain.

However, several studies demonstrated an increase in Δ[HHb] as well as Δ[HbO_2_], suggesting that the balance between oxygen consumption and oxygen delivery in the neonatal brain differs from the adult brain. Factors that effect NVC include the signaling pathways responsible for dilating blood vessels, which may still be developing and hence alter the expected increase in CBF, and myelination which effects the latency of the response (Harris et al., 2011).

It has been suggested that the NVC mechanism in the neonatal brain is not yet fully established and can lead to the differing response observed compared to adults, where NVC is well-established (Jasdzewski et al., 2003). The rapid developmental changes occurring in the newborn brain may effect the coupling between neural activity and blood flow, so fNIRS measurements may reflect the altered functional coupling of the brain (Kozberg and Hillman, 2016). The increase in oxygen consumption during neuronal activation may not always lead to overperfusion due to the immaturity of the vascular regulation in this cohort of subjects. Additionally, there may be a higher metabolic demand in these subjects compared to adults where metabolic demands in the neonate are still evolving that leads to a reversal of the balance between oxygen supply and consumption (Jasdzewski et al., 2003). Finally, it may be that NVC matures at different rates depending on the brain region, which may lead to varying responses dependent on functional tasks.

An increase in Δ[HHb] may also be related to venous dilation. Some studies, such as in Hoshi et al. (2000), observed an increase in Δ[HHb] not only with each subject but also within the same subject. An explanation for this may be related to increases of regional CBF which can lead to venous dilation and cause the increase in Δ[HHb] observed. It may be that cerebrovascular reactivity varies with developmental state (Hoshi et al., 2000).

Another possible explanation comes from a blood stealing effect, where regions surrounding the activated region receive reduced blood flow. Hence, an observed decrease in Δ[HbO_2_], as sometimes observed, or an increase in Δ[HHb] may be due to the activated region deeper in the brain “stealing” the blood flow from the fNIRS-measured volume or an activated region close to but not within the fNIRS-measured volume.

Kozberg et al. investigated the hemodynamic response in neonatal rats during electrical hindpaw stimulation reporting an increase in Δ[HHb] (Kozberg et al., 2013); this inverted response changed as the rats matured, developing to the characteristic hemodynamic response of an increase in Δ[HbO_2_] and a decrease in Δ[HHb]. Importantly, they reported increases in systemic blood pressure occurring during stimulation, with their magnitude dependent to the stimulation strength. These systemic blood pressure changes produce fluctuations in hemodynamics and oxygenation in the rat newborn brain that are exaggerated due to the underdeveloped cerebral autoregulation capacity. This acts as a significant confounding factor that can attenuate the hemodynamic response, invert it or even produce one in the absence of evoked neural activity. This physiological phenomenon and issue has been well described and discussed in adult functional activation studies with fNIRS as a major factor in producing false positives and false negatives (see recent review by Tachtsidis and Scholkmann, 2016).

Finally, the waking state of the newborn should be considered as this may affect the response seen to a stimulus. Four studies reported responses in awake newborns (Meek et al., 1998; Bartocci et al., 2000; Nissila et al., 2004; Kotilahti et al., 2005), nineteen studies reported responses in asleep newborns (see Table 1), one study reported responses in sedated newborns (Isobe et al., 2001) and the remainder of studies reported responses in a mixture of awake/quiet rest and asleep subjects. It is unclear how different arousal states can affect the neurovascular response. The study in the sedated newborns (Isobe et al., 2001) showed a slower hemodynamic response. Kotilahti et al. (2005) found a diminished response to an auditory stimulus when neonates were in quiet sleep compared to active sleep. Furthermore, Aslin (2012) has suggested that regional differences observed in sleeping neonates may disappear when arousal of the neonate increases and hemodynamic responses increase and could potentially override any regional differences seen.

### Study design and data analysis

The majority of fNIRS studies use a block paradigm, where periods of the experimental condition are alternated with periods of rest, and the changes in [HbO_2_] and [HHb] over the stimulation period are block-averaged to obtain a hemodynamic response. This repetition in the experimental condition has been demonstrated to reduce noise arising from uncorrelated trends (Yamada et al., 2012). Furthermore, it is not always possible to extract a functional response from only one trial due to insufficient signal-to-noise ratio (SNR) and motion artifacts (Scarpa et al., 2010). In some cases, a block design is not possible, for example, in pain studies, where the subject is presented with the pain stimulus only once. In this case, the amplitude of the hemodynamic response to such a stimulus is large in comparison to other stimuli such as touch, which enables a relatively clear response to be observed. However, such stimuli cause large systemic changes including heart rate and breathing rate which can lead to additional non-evoked physiological changes in the brain (Tachtsidis and Scholkmann, 2016). Care should also be taken when considering the number of trial repeats to perform; a recent infant fNIRS study showed a diminished cerebral response with increasing number of trials (Lloyd-Fox et al., 2010).

Many of the auditory studies presented here used more complicated paradigms such as subtle variations in speech to investigate language development in the neonatal brain. The various complexities in stimuli used makes the expected direction of response more difficult to interpret and compare between studies, which may be responsible for some of the non-typical responses observed. A review of the influence of experimental design on the hemodynamic response in infants has recently been discussed in Issard and Gervain (2018).

Data processing and analysis also varied across studies and may affect observed responses. One issue that is evident from the presented literature is the inconsistency in reporting all the NIRS parameters. Additionally, whilst some studies include Δ[HHb] in graphs showing hemodynamic changes, many neglect to discuss the results of Δ[HHb] or choose not to perform statistical analysis on this parameter. In order to better understand the typical hemodynamic response in neonates, it is imperative that studies report the results of both Δ[HbO_2_] and Δ[HHb] to allow a greater understanding of the behavior of these signals.

One reason authors preferentially report Δ[HbO_2_] is due to its repeatability across studies. Δ[HHb], in comparison, has a more heterogeneous behavior (Dravida et al., 2017) which may be due to its lower amplitude and the lower SNR of this parameter. Since fNIRS has the capability of measuring both Δ[HbO_2_] and Δ[HHb], it would be beneficial to report both parameters as this utilizes all the information available and provides a more comprehensive view of the response.

As well as study design, data analysis techniques used differ between studies. Pre- processing techniques are often used on fNIRS data, and consist of low-pass filtering (ranging from 0.25 to 1 Hz) to remove slow drifts and slow oscillations, and high-pass filtering (ranging from 0.01 to 0.05 Hz) to remove pulse artifacts and other high frequency noise. Many studies also removed stimulus epochs that contained movement artifacts or otherwise removed the movement artifact and interpolated the data, which were identified either visually (appearing as spikes in the data), via large standard deviation changes during the stimulus period or via monitoring of video footage of the infant. Smoothing of the data was also performed in some studies, for example using a moving-average window.

Extraction of the hemodynamic response also differed, with no standardized method yet established. A common method is to perform a block average of the data across epochs and to determine activation via comparison of the peak period against a baseline period before presentation of the stimulus. Often, data is presented as a group average, where results from all infants are averaged and presented. One issue with this method is the potential for the variation in Δ[HHb] to be masked by the averaging process; data should also be assessed on an individual basis to check for differences in Δ[HHb] response which may have a physiological meaning. An alternative approach employed by some studies is to use a general linear model (GLM) to extract the hemodynamic response, where the measured signal is explained in terms of a linear combination of the modeled response plus an error term. This is an increasingly used technique that is a standard method in fMRI data analysis. However, similar to fMRI, modeled responses are usually based on adult responses with a need for an infant HRF.

The most commonly used statistical techniques to assess activation were ANOVAs and student's *t*-tests. A review of statistical analysis in fNIRS can be found by Tak and Ye (2014).

### fNIRS instrumentation

There is a range of NIRS instrumentation that can be used to monitor cerebral hemodynamics, with the main methods comprising of continuous-wave systems, time-resolved systems, and frequency-domain systems. A review of these different modalities can be found in Scholkmann et al. (2014).

The wavelengths used, number of channels, and source-detector separation are all important in characterizing a system and are discussed briefly below.

#### Wavelength selection

NIRS systems require a minimum of two wavelengths to determine concentration changes of two unknowns: HbO_2_ and HHb. However, many systems use more than two wavelengths in order to reduce cross-talk by improving separability between the two absorption spectra of these chromophores. Cross-talk is defined here as a genuine change in one chromophore concentration inducing a spurious change in another chromophore concentration. This is especially important in functional activation studies where the focal nature of chromophore changes can lead to cross-talk due to incorrect pathlength assumptions (where differential pathlength rather than partial pathlength is used; Boas et al., 2004). The selection of wavelengths across the NIR region is important in determining the accuracy of concentration measurements derived (Strangman et al., 2003). Furthermore, optimum wavelength combinations are important to maximize SNR, as low SNR may mask chromophore concentration changes (Sato et al., 2004).

**Figure 8:**
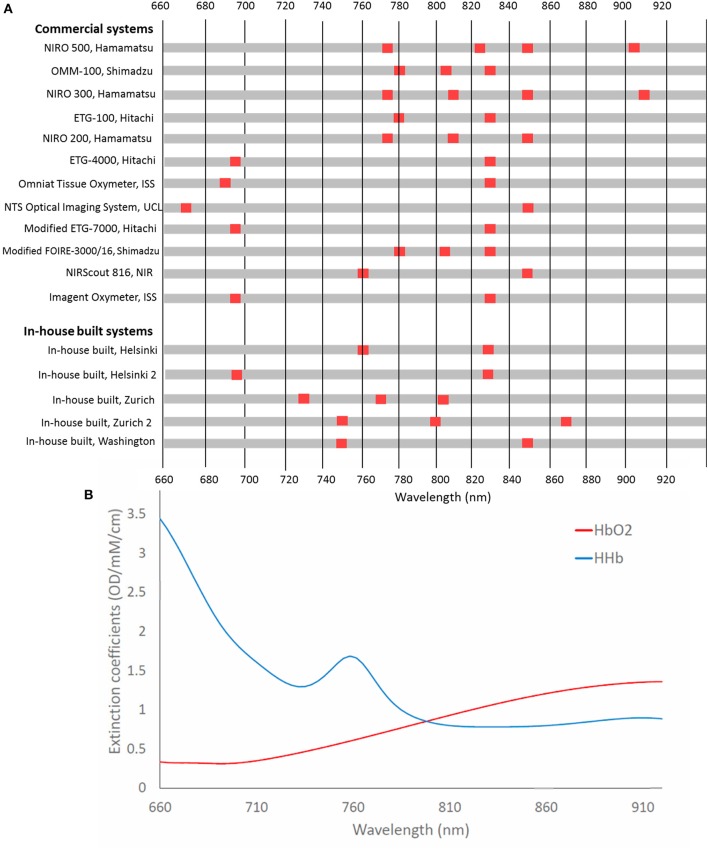
**(A)** Chart showing wavelengths used in NIRS systems used in functional studies of newborns <1 month of age. Systems are listed in chronological order from their first publication as used for this application. **(B)** Extinction spectra for HbO_2_ and HHb for the corresponding wavelengths.

Figure 8 summarizes the different wavelength combinations used in different NIRS instruments, with the number of wavelengths used for functional activation studies in newborns varying between two to four wavelengths, ranging from 670 nm up to 910 nm. Generally, systems use at least one wavelength above and below the isosbestic point (around 800 nm) to differentiate between signals sensitive to changes in Δ[HbO_2_] and in Δ[HHb].

Uludag et al. (2004) used model-based estimates of cross-talk and separability to assess the best wavelengths to use to accurately assess concentration changes of HbO_2_ and HHb in the adult head. They found that if both wavelengths are >780 nm, then cross-talk is high. Additionally, their theoretical optimum wavelengths, with one wavelength below 720 nm and the other wavelength >730 nm, were in contradiction to wavelengths used in commercial systems, although it should be noted that this study was based on the adult head. Additionally, Boas et al. (2009) identified optimum wavelengths when one wavelength is <710 nm and the other is above 830 nm. It is expected that selected wavelengths with a large difference in the absorption coefficients between HbO_2_ and HHb might yield more reliable concentrations hence similar results might be expected for the newborn. Additional recommended wavelength combinations have been summarized by Scholkmann et al. with the combination 780 and 830 nm generally shown to be more susceptible to cross-talk (Scholkmann et al., 2014). It is well acknowledged that more near-infrared wavelengths lead to a better separation and quantification of the changes in chromophore tissue concentrations (Arifler et al., 2015).

Looking only at the wavelength selection independent of other factors, studies showing a variation in Δ[HHb] tended to use wavelengths outside the recommended range. Of the three studies that utilized three wavelengths, 1/3 showed an increased Δ[HHb] with one not reporting Δ[HHb] and the remaining study reporting a negative Δ[HHb]. Likewise, use of 780 and 830 nm had a high proportion of varied (4/6) compared to unreported (2/6) Δ[HHb]. A reversal of response has previously been reported when investigating optimal wavelength combinations in adults (Uludag et al., 2004). This was found using wavelength combination 790 and 920 nm; no newborn studies using these two wavelengths were identified although this highlights the importance of appropriate wavelength selection for accurate concentration quantification in fNIRS. Sub-optimum wavelength selection is likely to affect Δ[HHb] results more than Δ[HbO_2_] due to the lower SNR of this parameter.

#### Probe placement and number of channels

The number of sources and detectors of a system determines the coverage on the head or region of interest. Sources and detectors can be arranged in such a way that multiple sources can reach multiple detectors, thereby increasing the number of available channels. An increased coverage reduces the uncertainty over which cortical region is being monitored, however, this can increase the chance of optical cross-talk at detectors, where the origin of the light is uncertain due to multiple sources.

The number of channels used varies, with five studies using 1 channel, nine studies using between 2 and 10 channels, twenty five studies using between 11 and 24 channels and seven studies using over 25 channels.

Probe placement is crucial in studies monitoring brain activity in a specific cortical region, as incorrect placement may lead to the activated tissue volume of interest not being interrogated. Kleinschmidt et al. (1996) performed simultaneous fMRI and fNIRS measurements in healthy adults and found no fNIRS-HHb response when NIRS probe positions were 1–2 cm away from the region of activation. Most studies determined probe placement using the 10–20 system, with probes placed with respect to anatomical landmarks on the head. This enables more reliable placement between subjects, where head size and shape may vary and aids in standardization of probe placement. However, the rapidly maturing brain of newborns adds to the difficulty in accurate placement with variation occurring amongst individuals between external landmarks and internal brain structures (Kabdebon et al., 2014).

Multi-channel systems have the advantage of being able to cover a larger area of the cortex; since the underlying brain structure of newborns is unknown on an individual basis, single channels may miss the region of activation. It has additionally been shown in adult studies that reliability of fNIRS measurements is improved through averaging over several channels (Wiggins et al., 2016). Furthermore, multiple regions of the brain can be monitored simultaneously. This can be beneficial in identifying stimulus-specific responses; by monitoring regions where no activation is present, non-stimulus related responses (such as those due to arousal state of the newborn) can be ruled out (Aslin, 2012).

**Figure 9:**
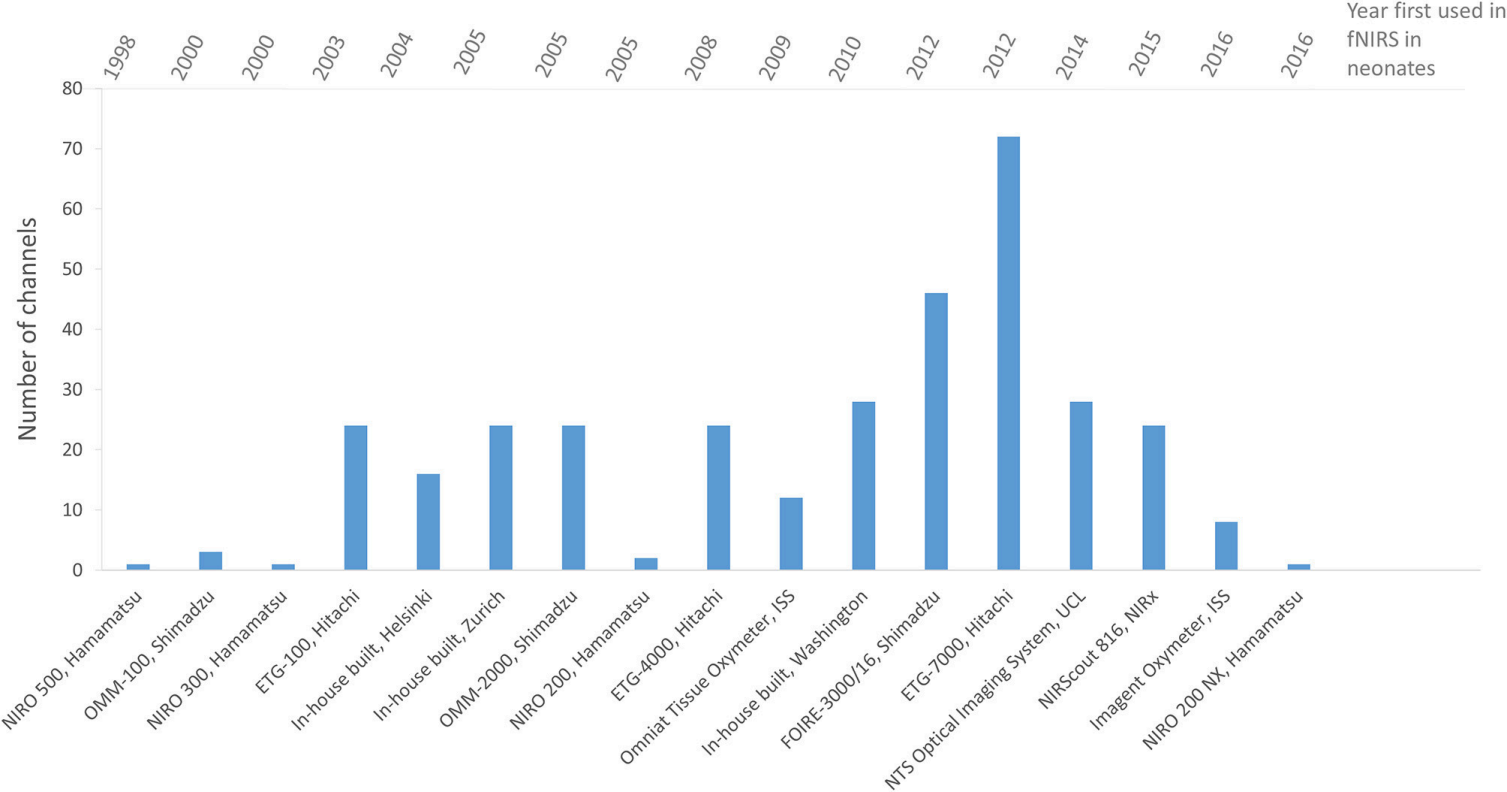
Chart showing the number of channels used in fNIRS systems for functional studies of newborns <1 month of age. Systems are listed in chronological order as presented in the literature.

An increase in the number of sources, detectors and channels used in fNIRS studies can generally be seen over the years as the technology develops, indicating the preference for a wider head coverage as researchers keep up with instrumentation developments. Figure 9 summarizes the number of channels used in fNIRS studies in newborns showing in general an increase in channels with date of publication.

#### Source-detector separations

Different source-detector separations interrogate different depths of tissue, with short separations sampling more superficial layers and longer separations more likely to sample the brain. This effect is less significant in newborns compared to adults as the newborn skull thickness is around half of that of a typical adult. Hence, smaller source-detector separations can be used and still interrogate cerebral tissue. Source-detector separations of 2.5 cm have been shown to have sufficient depth penetration of around 1 cm, which includes the cerebral cortex in neonates (Boas et al., 2009).

The source-detector separation as well as placement of the probes is important for determining the sampling region, as even a slight difference in this sampling region can lead to differences in results. One example is from Gervain et al. (2011) who performed a study similar to their 2008 study on speech structure in the neonatal brain (Gervain et al., 2008) using a shorter source- detector separation of 2 cm compared to the 3 cm originally used. They found that, whilst the 2 cm channel was able to identify the overall auditory activation, it failed to register more subtle results seen at 3 cm, where no response was seen in the frontal region at 2 cm and differences between two grammar conditions were not seen. This shows that care should be taken when interpreting fNIRS results as absence of functional activation is not conclusive of no activation; smaller source-detector separations may not be able to detect activation where longer source-detector separations are able to. This may preferentially affect Δ[HHb] due to the spatially smaller change in this parameter compared to Δ[HbO_2_] during functional activation (Strangman et al., 2003), making a longer separation more likely to sample the activated region and detect changes due to Δ[HHb].

Source-detector separations vary across instrumentation, ranging from 1 to 5.6 cm. An optimum source-detector separation enables monitoring of the deeper tissue in the brain whilst still maintaining a high SNR and hence, increases the likelihood of stimulus-induced cerebral changes being detected. The anatomy of the neonate as well as the age should be taken into account when determining source-detector separation as this will affect the interrogation volume and largely differs from those used on adult heads. Light source power will also affect the penetration depth and quality of the signal. Hence, optimal source-detector separations may vary depending on instrumentation and age of subject.

Finally, some studies have employed regression techniques in order to remove scalp interference from NIRS measurements and reveal only cerebral changes. Use of multiple separations are able to provide depth discrimination and superficial effects can be removed from longer channels through various signal processing techniques (Tak and Ye, 2014). This has been shown to increase SNR in the cerebral response. These techniques are becoming more prevalent in fNIRS studies (Gagnon et al., 2011).

As can be seen from Figures 8, 9, there is no standardization in the instrumentation used for functional studies in newborns, with varying wavelength combinations, source-detector separations and number of channels.

## Conclusion

This review has collated and summarized the studies to date that have utilized fNIRS in term neonates <1 month of age. In total 46 papers were found, with some studies investigating more than one stimulus, resulting in a total of 51 presented responses. The majority of papers identified an increase in Δ[HbO_2_]. However, a large proportion of papers do not report Δ[HHb] (only 24/51 papers explicitly discussed the direction of Δ[HHb]). Of the papers that do report the direction of Δ[HHb], the majority show a decrease in Δ[HHb], with 17/24 papers stating a decrease or no change in this variable. This is in contrast to a recent review by Issard and Gervain (2018) who looked at a broader population of infants including newborns. They identified variable hemodynamic responses, with a canonical response of an increase in Δ[HbO_2_] and decrease in Δ[HHb], or an inverted response of a decrease in Δ[HbO_2_] and increase in Δ[HHb], with some studies also reporting changes in the same direction.

The typical hemodynamic response in newborns is not well-established; there is a desire to identify a typical response in a healthy infant brain such that future work could enable detection of abnormal developmental patterns in neonates with brain injury such as HIE.

Potential reasons for the discrepancy in Δ[HHb] in newborns could be due to the differing physiology in this cohort, or instrumentation and analysis differences across studies. Liao et al. (2010) suggest that the ambiguity in Δ[HHb] response may be resolved by using a tightly controlled age range, as differences in age may reveal the maturation of the developing newborn brain if discrepancies are due to this. Additionally, locations of source-detector positions and scalp interference may contribute to the observed heterogeneity.

There has been a vast improvement in instrumentational developments since the first study in 1998, with multi-channel systems becoming increasingly common, and the emergence of diffuse optical tomography systems. Furthermore, cap designs have improved enabling better coupling with the head, and the increase in multi-modal imaging enables more robust analysis of functional activation. New developments in fNIRS technology will further allow us to monitor both hemodynamic and metabolic responses during newborn functional activation, with broadband instruments able to measure metabolic marker, cytochrome-c-oxidase (Bale et al., 2014). This metabolic marker has shown to have increased brain specificity (Kolyva et al., 2014; de Roever et al., 2017), enabling us to investigate hemodynamic metabolic coupling during neuronal activity (Bale et al., 2016; Siddiqui et al., 2017). Finally, developments in analysis techniques, such as short-separation techniques to remove scalp effects (Gagnon et al., 2011) enable improved data analysis.

In order to address the heterogeneity in the HHb response, we suggest here a set of guidelines which may help to identify and explain the behavior of this parameter. We suggest that future studies using fNIRS in newborns should:

- Report both hemoglobin parameters (Δ[HbO_2_] and Δ[HHb]) as these provide a more complete picture of the hemodynamic response than just Δ[HbO_2_] on its own
- Show the full time-course of the hemodynamic response, where latency of the response may help inform on brain maturity
- Attempt to differentiate between sedated, asleep and awake infants in the results as the different arousal states may confound the hemodynamic response
- Report on any individual cases of a varied hemodynamic response which may be masked at the group level but still have physiological meaning

In addition to the above, there is a need for (i) appropriate statistical framework for inference of newborn functional activation (such as the development of a newborn HRF for GLM analysis) and (ii) multimodal measurements that include systemic variables such as heart rate and blood pressure to identify confounding factors of the newborn brain hemodynamic response.

There is great potential for fNIRS to be utilized to monitor newborns with brain injury at the cotside, and provide valuable clinical information that could aid with clinical care. There is therefore a positive future in the field of monitoring newborn responses to stimuli, with continuing improvements in instrumentation and analysis.

## Author contributions

IdR, GB, and IT wrote the first draft of the manuscript. IdR, GB, SM, JM, NR, and IT participated in the analysis, drafting, and revising of the manuscript.

### Conflict of interest statement

The authors declare that the research was conducted in the absence of any commercial or financial relationships that could be construed as a potential conflict of interest.
